# Geometric morphometric analysis of an ontogenetic cranial series of the Permian dicynodont *Diictodon feliceps*

**DOI:** 10.1098/rspb.2024.0626

**Published:** 2024-07-31

**Authors:** Caitlin Rabe, Jesús Marugán-Lobón, Roger M. H. Smith, Anusuya Chinsamy

**Affiliations:** ^1^ Department of Biological Sciences, University of Cape Town, Cape Town 7700, South Africa; ^2^ Universidad Autonoma de Madrid, Madrid 28049, Spain; ^3^ Evolutionary Studies Institute, University of the Witwatersrand, Johannesburg, South Africa; ^4^ Karoo Palaeontology, Iziko South African Museum, Cape Town 7700, South Africa

**Keywords:** dicynodont, *Diictodon*, ontogeny, geometric morphometry, allometry

## Abstract

The Karoo Basin of South Africa is renowned for its abundance and diversity of therapsid fossils. Among the most ubiquitous and persistent of the Permian fauna is the small herbivorous dicynodont *Diictodon feliceps*. Intraspecific variation in *Diictodon* is historically confounding, and while ontogeny is frequently cited as a potential source of variation, observable developmental changes have never been calibrated. The present study revisits this issue, comparing three-dimensional landmark configurations of 82 *Diictodon* crania to investigate the association between shape, size and dimorphism. Beyond the statistically significant relationship between shape and allometry, our results determine the shape differences between juvenile and adult skulls of *Diictodon*, aligned with common craniofacial features documented in other tetrapod taxa. Functionally, these changes are attributed to development of the jaw musculature for feeding on larger, tougher plant matter during later ontogeny. Cranial morphological variation owing to sexual dimorphism is negligible, but distinct differences are noted in the allometric trajectories of each morphotype. A component of non-allometric variation cannot be accounted for, and we propose that this represents natural variation, rather than an artefact of taphonomic deformation.

## 1. Introduction

The dicynodontia were a diverse group of specialized herbivorous tetrapods, abundantly distributed worldwide, that ranged from the middle Permian across the Permo-Triassic boundary and into the Late Triassic [1–6]. Their persistence through the biodiversity crises of the late Permian suggests that they were highly adaptable with cranial and postcranial adaptations to exploit a diverse range of ecological niches [2,7–19].

Among the most abundant dicynodont genera in South Africa’s Karoo Basin is the small and widespread pylaecephalid, *Diictodon*, which has been found within the terminal chambers of helical burrow systems [10,17,20,21]. Fossils of *Diictodon feliceps* have been identified in China and Zambia but occur most abundantly in the late Permian lower Beaufort Group succession in the Karoo Basin of South Africa (approx. 260–252 Ma) [3,22–24]. Here, *Diictodon* persists through four Permian Karoo tetrapod assemblage zones (AZs)—an approximate temporal range of 12 Myr: they first appear in the Capitanian within the *Tapinocephalus* AZ, reach peak abundance in the *Endothiodon* AZ, subsequently declining in abundance in the late Permian before disappearing from the record in the Changhsingian within the upper *Daptocephalus* AZ [25–27]. The abundance of *Diictodon* in the fossiliferous beds of the Karoo basin represents 72% of the vertebrate fossils of the *Endothiodon* AZ [14,28], making it the most abundant genus of Dicynodontia in this biozone.

### (a) Intraspecific variation in *Diictodon*

Taxonomic inflation in the early twentieth century saw the emergence of over 50 synonyms of the type species *D. feliceps* but extensive revision indicates that the genus is likely monospecific [21,24,25,29,30]. However, the observation of intraspecific variation, particularly regarding the presence of both tusked and tuskless morphotypes, has continued to raise questions about the nature of variation in *Diictodon* [23,31–33]. Recent investigations have shown that while some discrete variation between morphotypes can be observed, such as the development of a bony pineal boss in tusked specimens, the difference in linear cranial proportions between the two morphotypes is negligible [21,30]. Based on the overall morphological similarity of the morphotypes, and their comparable co-occurrence within the sampled stratigraphic horizons, the currently held theory suggests that *Diictodon* tusks are an example of a sexually dimorphic armament [21,30]. In contrast, Laab *et al*. [34] suggest there may be reasonable evidence that these morphotypes represent distinct species, after tomographic analysis of the inner ear found there to be significant structural differences between a tusked and tuskless specimen.

An area of research that is limited, both in *Diictodon* and more broadly among non-mammalian therapsids, is ontogenetic morphological variation. While describing and understanding ontogenetic growth is a common outcome of palaeohistological research on dicynodonts (e.g. *Wadiasaurus* [35]; *Placerias* [36,37]; *Lystrosaurus* [7,11,15,16,38,39]), there are few descriptions of gross anatomical variation through ontogeny. In their broad study of synapsid cranial allometry, Krone *et al*. [40] compared the ontogenetic trajectory of *Diictodon* with the interspecific allometric pattern recovered across Anomodontia. Their findings indicate that *Diictodon*’s ontogenetic growth is parallel to that of the interspecific allometric trend for anomodonts but the authors do not describe this ontogenetic variation in detail [40]. Additionally, Kammerer *et al*. [41] used a sample of *Diictodon* to test the effects of taphonomic deformation on intraspecific morphospace analyses. The results of this study found a weak relationship between size class and shape in undeformed specimens, which was further overprinted by the effects of deformation in a larger, mixed sample [41]. Considering these findings, cranial variation in *Diictodon,* particularly as it pertains to dimorphism and ontogeny, emerges as a complex issue that warrants further investigation. The aim of this study is to investigate and quantify the contribution of ontogeny to intraspecific variation within the cranial morphospace of *Diictodon*, and to examine its interactions with dimorphism.

## 2. Methods

### (a) Geometric morphometry

Traditional morphological comparisons quantify the differences in homologous structures between organisms using simple linear measurements and their associated ratios. The linear nature of these measurements is such that analysis of information about an object’s shape, independent of its size, is limited [42,43]. An alternative approach is geometric morphometry, which allows for a more detailed characterization of shape variation. Within this framework, linear measurements are replaced by a constellation of Cartesian landmark points that are configured to capture information about an object’s shape [44,45]. Analysis of shape between objects compares these landmark configurations through superimposition under the generalized Procrustes least squares criterion. This process translates, rotates and scales all the configurations in a sample, without disrupting their internal integrity, to minimize the distance between corresponding landmarks and remove non-shape variation among specimens [46]. The superposition, or alignment, is performed using the mean configuration as the reference, by consensus, and any remaining mismatch that we observe between the configurations represents real shape differences between specimens [42,43]. Analysis within this framework enables the construction of comprehensive models and graphical interpretations of shape variation that lends itself to morphologically meaningful illustrations [43,47].

### (b) Data collection

**Figure 1.**
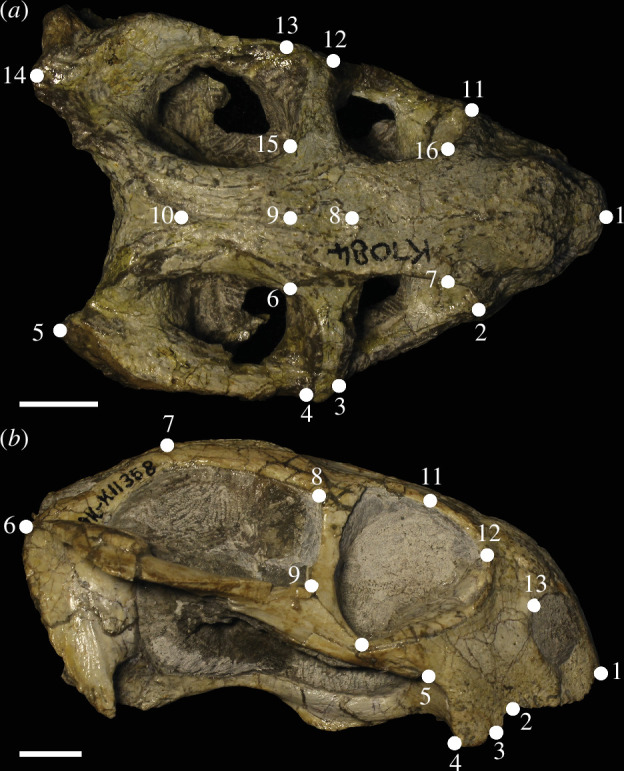
Dorsal (*a*) and lateral (*b*) view of *Diictodon* skull showing landmark configurations. Scale bars equal to 10 mm. For descriptions of landmarks, see electronic supplementary material, table S1. (*a*) SAM-PK-K7084; (*b*) SAM-PK-K11358.

This study examined over 1500 specimens of *Diictodon* crania from the Karoo palaeontology collections of Iziko South African Museum in Cape Town. The entire collection was examined, and specimens were selected for the study on the condition that a substantial portion of the cranial morphology was visible in either the dorsal or lateral view. Specimens that are fragmentary, poorly prepared or exhibit asymmetrical taphonomic deformation were excluded. The resulting sample (*n* = 82) represents approximately 6% of the collection and even these selected specimens exhibit some slight deformation. This highlights the inherent difficulties of working with fossil material and emphasizes the importance of methodologies that reduce the influence of taphonomy to optimize the available sample size. Two landmark configurations were devised in accordance with the most common modes of preservation that were observed in the Iziko collection—dorsoventral compression (*n* = 38) and mediolateral compression (*n* = 51) (figure 1; electronic supplementary material, table S1 and figure S1). To reduce the noise caused by taphonomic deformation, specimens with similar preservation were grouped and analysed together. Specimens ranged in size from 1.8 to 13.5 cm and were sorted into incremental size classes by skull length (SL; from posterior surface of basiocciptal condyle to snout tip), and landmark data were digitized using a MicroScribe G2X three-dimensional digitizer. Landmark configurations for each specimen were digitized three times and then averaged to reduce digitization error.

While some specimens were complete, most specimens were deformed or damaged to some degree which resulted in absent landmarks, and skulls that could only be digitized on one side. To maximize the sample size for this study and to optimize the landmark configuration, missing landmarks were reconstructed prior to morphometric analysis of the data. In cases where one-half of the skull was not viable for digitization, the bilaterally symmetric landmarks from the undeformed half were used to reconstruct the missing side using sagittal landmarks to establish a line of symmetry [46,48,49]. In mediolaterally and dorsoventrally compressed specimens where landmarks were absent on both sides of the skull, the position of missing landmarks was estimated through mean substitution [50–52]. This approach to missing landmark estimation calculates the mean observation for each variable from all the observations in a dataset and then substitutes the mean observation for each of the missing values [49,53]. Missing landmark estimations were performed in R, using a script developed by Sergio Nebreda in the Unit of Palaeontology at the Universidad Autónoma de Madrid, and in Morpheus [54] for the reflection and mean substitution of landmarks, respectively. All subsequent morphometric analyses were conducted using MorphoJ [55].

While incremental size classes are functionally useful for allometrically organizing specimens, they are not inherently ontogenetically informative. As ontogenetic studies on *Diictodon* are limited, the ontogenetic categorization used here derives from Ray & Chinsamy’s [56] histological analysis of postcrania. From bone microstructure and histological analyses of 13 specimens of *Diictodon*, Ray & Chinsamy described four distinct ontogenetic stages based on an estimation of body mass as a percentage of adult size [56]: early juveniles (<60% adult size), late juveniles (60–70% adult size), sub-adults (70–90% adult size) and adults (>90% adult). In the present study, 12 of the 13 specimens used in the previous study were re-examined to measure their SL, in accordance with our methodology. The SLs associated with the specimens used by Ray & Chinsamy [56] correlate with their designated ontogenetic stages as follows: early juveniles (SL < 9 cm), late juveniles (SL = 9.5 cm), sub-adults (SL = 10–12 cm) and adults (SL > 12 cm). As the present study encompasses a larger range of skull sizes, we included a neonate categorization, which denotes the age prior to tusk eruption, and we have expanded the above ontogenetic stages to optimize the spatial analysis of the data. It should be noted that accurately classifying the morphotype of small specimens is inherently difficult without the use of scanning technology to determine the presence of potentially unerupted tusks. We have established a threshold of between 5 and 6 cm SL below which there are currently no specimens with tusks. As such, for the purposes of this paper, we assert that below this threshold the morphotype cannot be diagnosed with confidence.

Hence, the ontogenetic stages used in this study to explore the dataset are neonate (SL = 1–6 cm; *n* = 8), early juvenile (SL = 6–9 cm; *n* = 38), late juvenile (SL = 9–10 cm; *n* = 13), sub-adults (SL = 10–12 cm; *n* = 18) and adults (SL > 12 cm; *n* = 6). It should be noted that none of the specimens examined by Ray & Chinsamy [56] had associated crania that were viable to be digitized for this study.

### (c) Shape analytical framework

Landmark configurations were superimposed through generalized Procrustes analysis [57,58] and all subsequent statistical evaluations were performed on the transformed Procrustes shape coordinates. The multidimensional Procrustes shape variation was explored through principal component analysis (PCA)—a common multivariate method used to statistically summarize the main vectors of shape variation between configurations.

Centroid size (CS) is the measure of size in morphometric studies and can be described as the square root of the sum of squared distances of all the landmark points for a specimen from their centroid [43]. Multivariate regression analyses on CS were performed on both dorsal and lateral sets of Procrustes coordinates to quantify the ontogenetic allometry on the observed shape variation, which should align with the categorized stages. A permutation test of 10 000 replicates was used to test the null hypothesis that shape variation is independent of size [59].

There are several potential sources of non-allometric variation in *Diictodon*—sexual dimorphism, biogeographic distribution, taphonomic modification, geological age and natural population variation. Most of the specimens sampled in this study were preserved in strata assigned to the Endothiodon AZ (*n* = 70), with few representatives from other stratigraphic intervals—*Daptocephalus* AZ (*n* = 5), *Cistecephalus* AZ (*n* = 5) and *Tapinocephalus* AZ (*n* = 1)—which suggests that the influence of stratigraphic age is limited. The *Endothiodon* AZ is constrained to ~260–258 Ma [14]. Furthermore, in their broad examination of *Diictodon*, Kammerer *et al*. (2020) found there to be limited evidence of stratigraphic variation in the sample but it is noteworthy that the impact of taphonomic deformation in their study may have overprinted any stratigraphic signal. Similarly, most sampled specimens originate from two geographic regions: Beaufort West (*n* = 34) and Fraserburg (*n* = 37), approximately 100 km apart, with fewer representatives from GraaffReinet (*n* = 8) and Murraysburg (*n* = 2). The distance between the two furthest localities, Fraserburg and Graaff-Reinet, is approximately 288 km. Using regression residuals (i.e. shape variation devoid of allometry [47]), we examined shape variation in the dorsal and lateral datasets to determine whether the remaining non-allometric variation could be attributed to dimorphism, geography or taphonomy. Both the Procrustes shape data and the allometric residuals were submitted to a discriminant function analysis (DFA) to test for a signal of sexual dimorphism in the structure of shape data. Allometric residuals were further explored through regression on morphotype, coded as a binary variable. For all analyses relating to sexual dimorphism, specimens with SL smaller than 6 cm were excluded.

While studies show that specimens that exhibit the same type of deformation cluster together [41,60], warranting the separation of lateral and dorsal samples, there remains a spectrum of deformation within each sample. For example, specimens that are dorsoventrally compressed were analysed together in the dorsal sample but this includes specimens that are almost completely flattened, as well as specimens that are only slightly flattened. Hence, if the degree of vertical compaction is the dominant taphonomic factor that modifies the shape of the skulls, then this variation could theoretically be removed by eliminating the measurements of skull depth in the dorsal configuration. To investigate how the degree of taphonomic deformation, namely dorsoventral and mediolateral compression, affects the two samples, we removed the third dimension from the landmark data. In the case that the degree of compression is the main source of taphonomic variation, we would expect that when this dimension is removed there will be a proportional decrease in the amount of residual variation.

## 3. Results

### (a) Cranial variation and allometry

**Figure 2.**
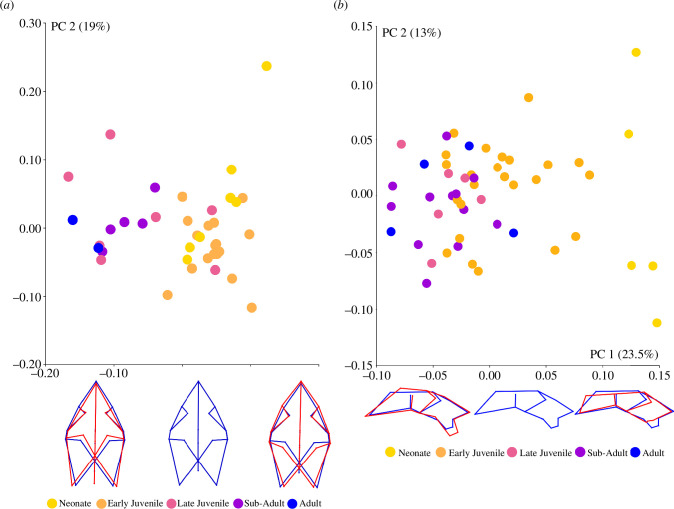
(*a*) PCA biplot of PC1 and PC2 for dorsal landmark configuration of *Diictodon*. Changes in shape (red) from the consensus (blue) are illustrated with wire-frame representations of the landmark configuration and represent (from left to right) the shape transition along PC1. (*b*) PCA biplot of PC1 and PC2 for lateral landmark configuration of *Diictodon*. Changes in shape (red) from the consensus (blue) are illustrated with wire-frame representations of the landmark configuration and represent (from left to right) the shape transition along PC1.

In the PCA for the dorsal landmark configuration, principal components 1–2 accounted for 48% of the cumulative variance in the sample (figure 2). In PC1, shape variation depicts skulls with a proportionally longer or shorter anterior region (i.e. the region of the skull anterior to the postorbital bars) that can be mediolaterally wider or narrower. The width of the intertemporal is conserved, but the parietal region also changes mediolaterally. The orbits are either smaller or larger, and when the latter occurs, it is in part facilitated by the lateral and posterior displacement of the postorbital bars. The squamosal processes can be closer together or further apart, with ventral or dorsal displacement, relative to the consensus shape. Accordingly, on the positive side of PC1 scores, the overall dorsal profile depicts a relatively wider skull with enlarged orbits, smaller temporal fenestrae and a proportionally larger anterior (figure 2).

There is considerable overlap between ontogenetic stages. Importantly, however, the specimens are ordered along PC1 in terms of size, such that relatively smaller specimens (neonates and early juveniles) occupy the positive values, and larger specimens (late juveniles, sub-adults and adults) occupy the negative values. The neonate and sub-adult specimens are more conserved in their variation along PC1 than the other stages, with the late juvenile specimens showing the greatest variation in their distribution along PC1. Specimens do not show distinct clusters or ordering along PC2.

The regression of the Procrustes shape coordinates of this skull view on CS captures a significant association between shape variation and allometric growth in the sample, accounting for 15.1% of the observed variance (*R* ^2^ = 0.151; *p*‐value < 0.0001).

In the PCA for the full set of lateral crania (figure 2), components 1–2 account for 36.5% of cumulative variance. In PC1, shape variation associated with positive values (i.e. particularly for the younger specimens) show a dorsoventrally shorter and less pronounced caniniform process relative to the palatal rim, a larger orbit and maxilla and premaxilla that are reduced both anteroposteriorly and dorsoventrally. The lateral profile associated with positive values depicts a flatter skull with a proportionally longer anterior, less elevated parietal region, reduced maxillary region and larger orbit. Like the dorsal dataset, there is some overlap between specimens of different sizes in morphospace (figure 2). Neonate specimens form a distinct cluster along PC1, although the juvenile, sub-adult and adult specimens overlap. In general, smaller specimens occupy the positive values, whereas larger specimens occupy the more negative values.

Like the dorsal configuration, multivariate regression of the Procrustes shape coordinates on CS indicates that there is a significant proportion of variation that can be explained by allometric growth, accounting for 14.5% of the observed variance (*R* ^2^ = 0.145; *p*‐value < 0.0001). For both configurations, this suggests that there are sources of variation, besides ontogeny, that are responsible for most of the variation in the lateral configurations between specimens.

### (b) Allometry and sexual dimorphism

**Figure 3.**
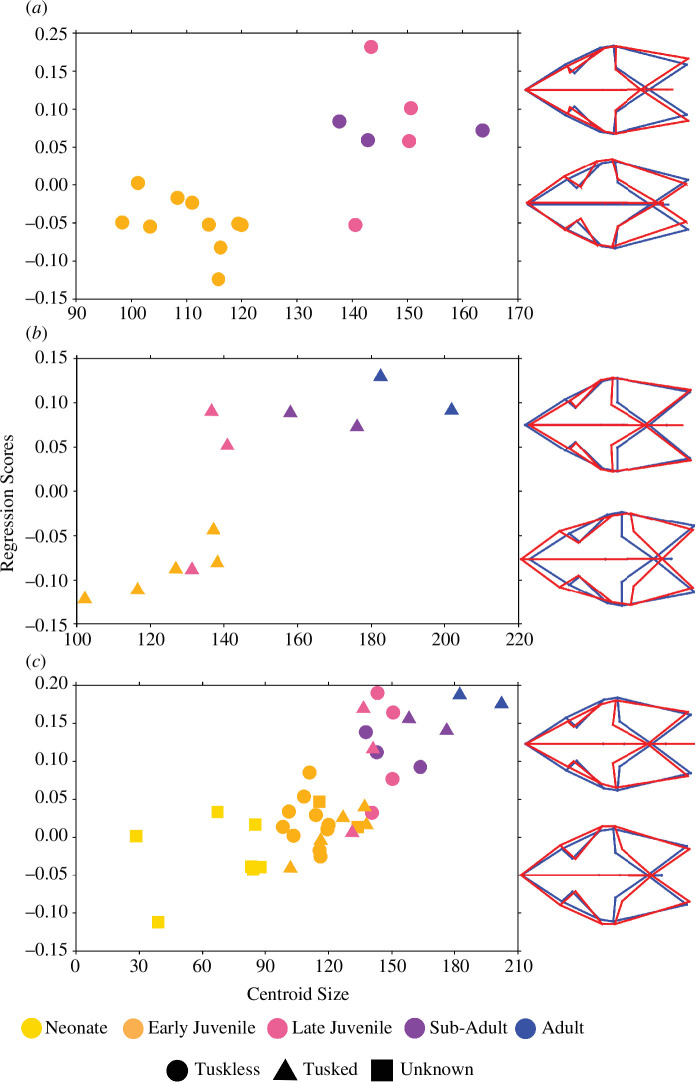
Multivariate regressions of Procrustes shape coordinates on CS for the (*a*) tuskless morphotype, (*b)* tusked morphotype and (*c*) combined population of the dorsal landmark configuration of *Diictodon*. Changes in shape (red) from the consensus (blue) are illustrated with wire-frame representations of the landmark configuration and represent the shape transition for smaller (bottom) and larger (top) specimens.

**Figure 4.**
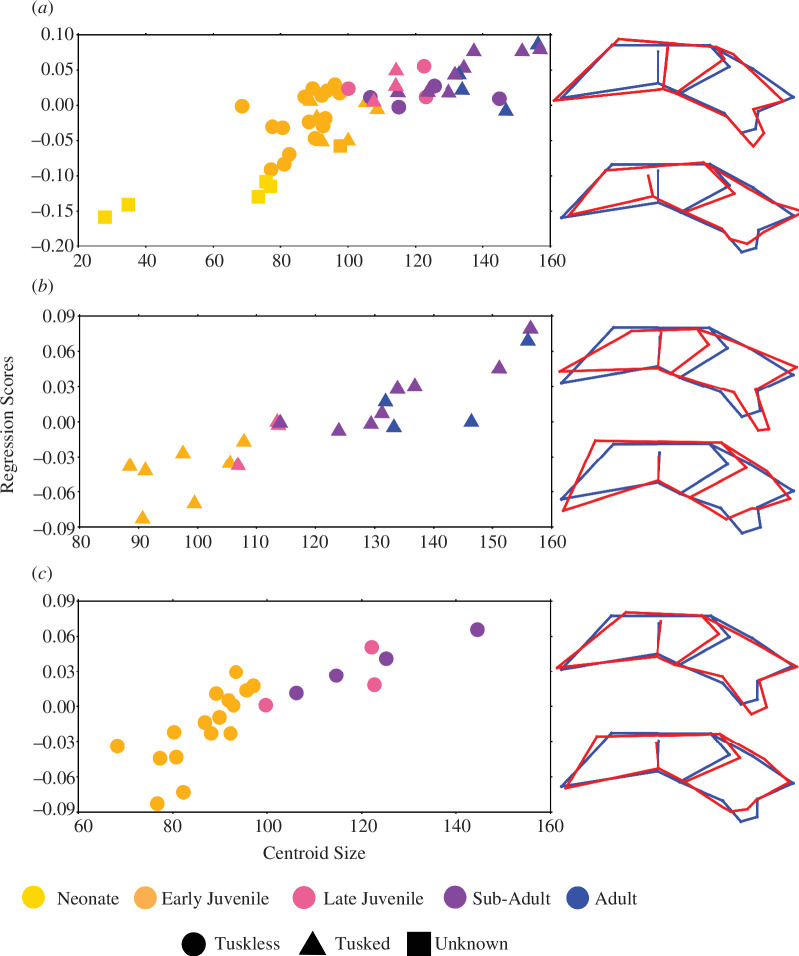
Multivariate regressions of Procrustes shape coordinates on CS for the (*a*) tuskless morphotype, (*b*) tusked morphotype and (*c*) combined population of the lateral landmark configuration of *Diictodon*. Changes in shape (red) from the consensus (blue) are illustrated with wire-frame representations of the landmark configuration and represent the shape transition for smaller (bottom) and larger (top) specimens.

Both morphotypes, tusked and tuskless, show a significant relationship between shape and CS in both the dorsal and lateral configurations at the 5% level (table 1). In both configurations, there is an apparent difference in the allometric trajectory between the morphotypes, although this is most distinct in the dorsal configuration (figure 3). In the dorsal configuration, the regression scores for tuskless specimens show a distinctly bimodal distribution that separates the sample into two groupings according to size, whereby the early juveniles form one group, and the late juveniles and sub-adults form the other (figure 3*a*). The tusked specimens also form two distinct groups of smaller and larger specimens, which are separated by differences in shape (figure 3*b*). Interestingly, both in tusked and tuskless specimens, there seems to be a notable shape change independent of size change (i.e. a shift in the shape-axis) that aligns with the transition between late juvenile and sub-adult stages, as determined by histology. For the lateral configuration, the allometries conform to a more linear trajectory (figure 4), suggesting that there is some unique shape information captured in the dorsal configuration that does not reflect in the lateral configuration.

**Table 1.** Results of the multivariate regression of shape on CS for *Diictodon* specimens of different morphotypes.

	population	*n*	% predicted	*p*‐value
**dorsal**	tusked	17	23.9523	0.0011
	tuskless	12	22.0754	0.0142
**lateral**	tusked	22	11.3276	0.0009
	tuskless	23	7.1989%	0.0483

Upon visual inspection of the vectors of shape change, both morphotypes undergo similar changes, such as the negative allometry of the orbit, transverse movement of the squamosal processes and a general ontogenetic shift from a wider, rounder profile with relatively larger anterior, to a more gracile profile with elongated posterior in the dorsal view (figures 3, 4). From these shape vectors, it appears that differences in ontogenetic shape variation between the morphotypes may be characterized more by the timing of emergence and proportions of shared features than by the development of functionally different morphologies. For example, both populations see a proportional shift from a dominant anterior region in early ontogeny to an elongated and widened posterior region later in life but this is more pronounced in the tusked population. In comparison, the tuskless population shows a more conserved change in shape through ontogeny, and relative to the tusked population appears to retain a more juvenile shape. Lateral shape vectors assist in visualizing the differential development of the caniniform process. Both morphotypes show a more pronounced caniniform through ontogeny, but in this case, the morphology is distinctly different. The ventral edge of the caniniform process in tusked specimens is uniformly flat, whereas tuskless specimens show a blade-like taper (figure 4). The tuskless morphotype maintains the width and blade-like shape of the caniniform throughout ontogeny, while the elongated caniniform processes in older tusked specimens are more slender relative to their width in early ontogeny.

The results of the DFA, tested against 1000 permutation rounds, indicate 37.93% (dorsal set; *p* = 0.6809) and 64.44% (lateral set; *p* = 0.0166) accuracy in the classification of morphotype based on the Procrustes shape data (table 2). The results of the DFA cross-validation (electronic supplementary material, figure S2) indicate considerable overlap between morphotypes, thus no shape distinction between them, but suggest that the lateral configuration contains more discriminating features.

**Table 2.** Results from the allometric and residual DFA for sexual dimorphism in *Diictodon* specimens.

		**morphotype**	classification		total	% of total
			correct	incorrect		
**allometric**	**dorsal**	tuskless	3	9	12	25
		tusked	8	9	17	47.06
	**lateral**	tuskless	15	8	23	65.22
		tusked	14	8	22	63.64
**residual**	**dorsal**	tuskless	5	7	12	41.67
		tusked	7	10	17	41.20
	**lateral**	tuskless	8	15	23	34.78
		tusked	7	15	22	31.82

### (c) Non-allometric cranial variation

PCA of dorsal and lateral regression residuals (electronic supplementary material, figure S3) reveals no distinct geographic clusters, and shows overlap between the two major localities, suggesting that the influence of geographic location on the remaining skull variation is minimal. Another PCA of regression residuals for both configurations (electronic supplementary material, figure S4) shows that the tusked and tuskless morphotypes overlap considerably in morphospace. Furthermore, the results of a regression of residual Procrustes coordinates on morphotype, which was constructed as a binary dummy variable, indicates that the separation of specimens by the presence/absence of tusks accounts for a negligible amount of the residual variation in the sample (lateral: *R* ^2^ = 0.0184, *p*‐value = 0.6876; dorsal: *R* ^2^ = 0.0336, *p*‐value = 0.03824). Using DFA to test for differences on the regression residuals, with 1000 permutations, morphotype was correctly identified with 41.38% (dorsal set; *p*‐value = 0.9545) and 33% (lateral set; *p*‐value = 0.8747) accuracy (table 2), showing considerable overlap between morphotypes, and thus, no discrimination is possible.

With the exclusion of stratigraphy, geography and sexual dimorphism, the remaining variation that we see in the sample may be taphonomic. The two-dimensional data showed less overall variation between specimens than in the three-dimensional set but there was an increase in the proportion of non-allometric variation, suggesting that the third dimension of the data does capture some information that is relevant to allometry (electronic supplementary material, table S2) and also indicating that the degree of ‘flatness’ between specimens, does not drastically influence the variation we see in the sample. In the lateral sample, overall variation between specimens increased when decreasing the dimensionality but there was a reduction in the proportion of residual, or non-allometric variation. While performing this experimental reduction in dimensionality can help us to assess the variation in skull height and depth that is caused by varied degrees of dorsoventral and mediolateral compression, this does not account for how the compression alters the spatial relationships between landmarks.

## 4. Discussion

The nature of morphological variation in the crania of *Diictodon* is historically confounding [21,30,33,41]. Comparisons of morphological dimensions and qualitative traits previously found much of the studied variation between specimens to be random, in the sense that it could not be statistically correlated with distinct and meaningful categories or groupings [30]. However, authors have continued to note the presence of observable differences in morphology across the population of *Diictodon,* with both ontogeny and dimorphism cited as potential sources of variation [21,40,41]. Our morphometric assessment of 82 *Diictodon* specimens showed significant and quantifiable changes in the shape of the cranium through ontogeny providing evidence that ontogenetic growth underlies part of the observable variation in crania among specimens. This supports the findings of Krone *et al*. [40] who also found a significant relationship between size and shape in the lateral skull profile of 39 *Diictodon* specimens.

Of all the skull regions that show distinctive ontogenetic changes, only the orbital region exhibits negative allometry with skull size. Negative allometry of the orbital region is frequently observed among tetrapods, where the orbit is proportionally larger in juveniles than it is in adults [61–63]. This corresponds to a well-recognized pattern known as ‘Haller’s Law’ which posits that smaller animals, in both a phylogenetic and developmental context, have proportionally larger eyes than larger animals in order to attain the same visual acuity [61,64]. Accordingly, in *Diictodon*, we show that the larger orbit in early ontogeny becomes proportionally smaller as the skull size increases. This is in part facilitated by the anteroposterior elongation of the skull, which produces a relatively narrower and shorter anterior region and decreases the length and width of the orbit. However, this change in relative orbit size may also come about as a secondary result of changes in the surrounding elements that are associated with the development of the jaw [62]. For example, lengthening of the snout is associated with anteroposterior shortening of the orbit which is an ontogenetic trend noted in both extinct and extant reptiles [65].

Furthermore, positive allometry of the snout or facial region relative to a decrease in the size of the braincase is also a long-noted interspecific trend in tetrapods related to growth, now referred to as the ‘cranial rule of evolutionary allometry’, or CREA [66,67]. This pattern of craniofacial allometry is also reflected in ontogeny, whereby adults tend to have proportionately longer snouts than juveniles [40,66,67]. Interestingly, in their broad analysis of Anomodontia, Krone *et al*. did not recover a significant shared allometric trend, noting that the general scheme of cranial variation in anomodonts can be characterized by the relative depth of the snout and elongation of the caniniform, rather than the length of the snout [40]. To expand on this work, our findings suggest that there may be a CREA-like pattern in *Diictodon,* as in the lateral configuration, we find not only a clear elongation of the snout through ontogeny but also the characteristic anomodont pattern of increased snout depth and a more pronounced caniniform, confirming that the latter is parallel to that of the allometric pattern they recovered for *Diictodon* [40].

The above trend of craniofacial shape change in the lateral view is difficult to determine in the dorsal configuration (figures 2 and 3), which appears to indicate that there is a relative decrease in the length of the snout through ontogeny. This disparity can be accounted for by the configuration itself, in which the extent of the snout was captured at the midpoint of the premaxilla to accommodate the number of specimens that were partially embedded, or broken, whereas the lateral configuration better captures the full extent of the snout in relation to the orbit and frontal regions. This issue highlights the importance of analysing specimens in as many views as possible in order to recover a comprehensive image of shape change, as well as the pervasive problem of taphonomic modification even when measures are taken to try and reduce the impact.

In light of these findings, *Diictodon* emerges as a potential outlier among the anomodonts. Recent studies suggest that the familiar mammalian pattern of CREA probably originated in Mammaliaformes rather than within non-mammalian synapsids [40,68]. Furthermore, more dedicated research into the ontogenetic allometries of *Diictodon* and other anomodonts may reveal a more basal origin of CREA or may support Krone’s assertion that beyond CREA there exists a more universal interspecific trend regarding the proportional constraints of the braincase and face in all tetrapods.

### (a) Ontogeny of the feeding mechanism influenced by diet

There is also an increase in the absolute and relative size of the postorbital region of *Diictodon* skulls through ontogeny, facilitated by an anteroposterior elongation of the squamosal processes, anterior movement of the postorbital bar and dorsoventral elevation of the skull roof in the frontal and preparietal regions. The resulting increase in the size of the temporal fenestrae has implications for muscle attachment and the development of the feeding mechanism. In dicynodonts, the temporal fenestrae are generally associated with the origin of jaw adductor muscles, which insert into the lateral surface of the mandible [1,69–71]. Reconstructions of dicynodont jaw musculature are based on comparisons with members of their extant phylogenetic bracket (extant reptiles and mammals), and through the observation of osteological correlates such as preserved muscle scars on the bone [18,72–77]. While there are variations in muscle attachment across genera, the generally accepted schema for the dicynodont jaw involves two main groups of muscles—external and internal adductors [1,78]. The external adductor muscles, responsible for closing the jaw during mastication, are postulated to have multiple sites of origin depending on the genus [1]. King [72] describes the external jaw adductor in two parts—the adductor externus lateralis (a.e.l.) and the adductor externus medialis (a.e.m.). King [72] describes the origin of the a.e.l. as the anterolateral surface of the zygoma, posterior to the postorbital bar, extending onto the lateral sheet of the squamosal process above the quadrate. The origin of the a.e.m. is described as the medial surface of the temporal fenestrae [72]. While no detailed muscular reconstruction exists for *Diictodon*, there are considerable similarities with the cranial plan of other dicynodonts that have been examined within the same phylogenetic bracket (e.g. *Eodicynodon* [1] and *Pristerodon* [18]) and these are generally consistent with King’s [72] reconstruction.

Thus, considering King’s [72] reconstruction of the jaw adductors, we see that the anteroposterior length of the temporal fenestrae and the dorsoventral height of the squamosal processes affect the available surface area for muscle attachment. Our results show that as *Diictodon* grew, the temporal fenestrae became larger and the squamosal processes elongated dorsoventrally, providing increased surface area for the attachment of the external adductor muscles. Additionally, there is a lateral migration of the squamosal processes during ontogeny, which increases the breadth of the temporal fenestrae. In her reconstruction, King [72] discusses the lateral flare of the zygoma, referred to herein simply as the squamosal process, in terms of its function. King [72] postulates that a more lateral origin site for the a.e.l. could balance the largely medial pull of the other adductor muscles. Alternatively, this lateral migration could be a response to orbital convergence which would laterally displace the postorbital bar and, consequently, the squamosal process [72]. Orbital convergence refers to the degree to which orbits are oriented in the same direction [64]. In our assessment of *Diictodon*’s development, we do not see evidence for the orbits becoming more convergent through ontogeny, which suggests that the lateral migration of the squamosal processes is not an indirect structural response to orbital convergence. While King’s suggestion of muscular balance cannot be ruled out, without a more detailed biomechanical reconstruction of the musculature of the jaw, we cannot confirm that this is the case.

The distinctive ‘beak-bite’ of dicynodonts such as *Diictodon*, in which the lower jaw is vertically elevated to close the mouth and plant material is sheared by the shearing action between the cutting edges of the dentary and maxilla, is largely achieved by the activation of the external adductors [18,73,79]. One clear consequence of the lateral expansion of the squamosal processes is the mediolateral enlargement of the temporal fenestrae that provides increased volume for the growth and development of the jaw adductor muscles, thus increasing the bite force. Additionally, the dorsoventral elongation of the squamosal could indicate a more dorsal origin of the external adductors, conferring a greater vertical force to the biting motion [73,74]. Studies of modern reptiles have shown that an increased bite force enables feeding on tough and brittle material [80–83]. As such, *Diictodon’s* cranial shifts through ontogeny towards larger temporal fenestrae, and larger more developed jaw adductors, may reflect an ontogenetic shift in diet towards more robust, tougher vegetation. This developmental trend towards greater muscle mass in the jaw is echoed within the phylogenetic trend for herbivorous tetrapods to develop relatively greater jaw adductors than their carnivorous counterparts [84]. Additionally, the dorsoventral and anteroposterior lengthening of the snout would enable *Diictodon* to bite and process larger volumes of vegetation as they grew, allowing them to keep up with their growing energy requirements. If we consider previous suggestions that dicynodonts may have grubbed for subsurface rhizomes [85,86], then we can speculate that the ontogenetic shift we observe in *Diictodon* may have enabled older individuals to nuzzle and scratch into the subsoil to feed on more fibrous and tougher tubers and roots.

### (b) Sexual dimorphism

Most of the variation observed in *Diictodon* appears to be non-allometric. Operating under the currently held notion that *Diictodon* is monospecific when ontogeny is removed from the structure of the data, there are several remaining factors that could account for this variation: sexual dimorphism, taphonomy, biogeography, stratigraphy and natural population variation. Stratigraphy and geography can be discounted since almost all studied specimens originated from the same AZ, and PCA dispersion indicates that geographic location has little effect (electronic supplementary material, figure S3). Historically, *Diictodon* specimens have been classified as male or female based on the presence or absence of tusks, respectively [21]. Our results show that the variable presence/absence of tusks in *Diictodon* does not significantly affect the cranial morphospace of the population, with and without the influence of allometry, and accounts for a negligible amount of the overall observed variation. Highlighting this point is the DFA which shows that when examining skulls of similar size based purely on shape information, morphotype is likely to be misidentified more than half of the time.

However, when taking allometry into account the chances of correctly identifying the morphotype improve. In the case of the dorsal configuration, identification by morphotype is poor whether or not allometry is considered. This makes sense given that the most obvious and significant features associated with dimorphism—the caniniform and tusk—are not captured in the dorsal configuration. The DFA aids in showing that there are some observable dimorphic features associated with ontogeny, apparently largely restricted to the region of the caniniform but there is still considerable morphological overlap between morphotypes. This supports the findings of previous studies which have also found their samples of *Diictodon* to be morphometrically homogeneous, despite the variable occurrence of tusks [21,24].

While dimorphism does not significantly structure cranial morphospace, we do note that the allometries of each morphotype appear to differ. When the vectors of shape change are considered in the dorsal configuration, a pattern emerges where the tuskless adults appear to retain a more juvenile shape in relation to the shape changes observed in the tusked population (figures 4, 5). It is worth noting that Angielczyk & Sullivan [24] noted a similar pattern in which juvenile male *Diictodon*—classed as specimens with partially erupted tusks or pre-eruptive resorption pits—had palatal proportions that were more like adult tuskless (female) specimens. The current assignment of sex, based on the presence/absence of tusks, assumes that tusk eruption occurs within a conserved horizon (SL between 5 and 6 cm). However, if the development and eruption of tusks is a more plastic feature within this species, then we must consider the possibility that some tuskless specimens classified as female may contain developing tusks that have not yet erupted. If this is the case, then it is possible that some of the tuskless specimens identified in this sample are not female but are rather immature males with unerupted tusks. This would account for the odd allometric trajectory observed in the female population and highlights an important avenue for future work—using scanning technology to assess outwardly tuskless crania for the presence of unerupted tusks.

The dorsal configuration of the tuskless morphotype exhibits a distinctly bimodal distribution (figure 3). In contrast to the tusked specimens which show a distinct shift in shape from a conserved juvenile form to a conserved adult form through a steady progression of size, the tuskless population almost appears to have two separate allometries with divergent directions. It is possible that these differences between morphotypes indicate some kind of ontogenetic divergence by which males and females achieve different morphological phenotypes at different sizes and rates [87–90]. However, if this was the case, there is no sensible reason why a similar signal would not be uncovered in the lateral configuration. Hence it is more likely that this distinct divergence from linearity in the dorsal tuskless population is a result of misclassification of the sex of the studied specimens. It is worth noting that the apparent transition in shape in the dorsal specimens, which occurs relatively fast, as denoted by the strong shift in the *y*-axis, coincides with the histological transition between late juvenile and sub-adult specimens as defined by Ray & Chinsamy [56]. It should also be acknowledged that aside from the potential misclassification of sex, the results may be influenced by sampling bias in the tuskless population, where there is a notable gap in CS representation (figure 3). Despite this potential bias, it is still noteworthy that this shape transition appears to occur earlier in the tusked population, where we can see a mix of late juveniles interspersed with early juveniles. The growth pattern depicted in the dorsal configuration which indicates a ‘step’-like transition in shape is similar to reptilian growth trends, such as that seen in crocodiles.

Given our observed differences in the growth trajectories of *Diictodon*’s morphotypes, and taking into account that some traits appear to be correlated with presumed sex [21], we must consider why sexual signals at the population level are negligible. In the context of the absence of strong sexual dimorphism in fossil taxa, it may be difficult to separate a young male from an older female if their size is dimorphic, particularly given that the exact ages of fossil specimens are unknown [91]. This may also hold true for dicynodont species, in which sexual dimorphism is infrequently detected. In fact, for the last 20 years, the most consistent and frequently cited evidence for sexual dimorphism in dicynodontia has been the variable presence/absence of tusks in *Diictodon*. While previous histological analysis has shown that *Diictodon* has an indeterminate growth strategy [56], to date no growth curves have been constructed for the species. With a limited foundation for the parameters of growth, small sample sizes for each sex (necessitated by poor preservation/deformation of specimens), and an inability to control for age, the notion of sexual shape dimorphism in *Diictodon* remains cryptic. Going forward, the establishment of growth curves, along with estimation of size at sexual and somatic maturity, will be critical to refining our understanding of sexual dimorphism in this dicynodont, and CT scanning emerges as a necessity for the accurate diagnosis of morphotype.

### (c) Teasing apart taphonomy and morphology

Although our study was designed to minimize the impact of taphonomy on our data, it is still likely that some of the observed variation is an artefact of preservation, as observed by Kammerer *et al*. [41]. Given the three-dimensional nature of the morphometric data, we predicted that one potential source of taphonomic variation may be the variable degrees of either mediolateral or dorsoventral compression within the lateral and dorsal subsets. The dorsoventral compression of the dorsal specimens distorts the landmark points. If this proved to be a considerable source of variation, it would cast uncertainty on all the aspects of ontogenetic shape change related to the height of cranial elements. However, we showed that reducing the number of dimensions only decreased the proportion of residual variation in the lateral set, conversely showing a slight increase in the dorsal set. Thus, while the degree of compression may influence the variation between similarly deformed specimens, the impact of this is small. Bilaterally symmetrized cranial reconstructions should eliminate the asymmetrical component of deformation but there is likely a symmetrical component of deformation that remains uncorrected. If during deep burial and permineralization, the compaction of the fossil is uniaxial, causing the skull to stretch or shrink uniformly, then our reconstructions cannot eliminate this symmetrical deformation. Additionally, there is always a possibility that some aspects of ontogenetic shape change may coincide with shape variation related to deformation [92]. Hence, while the ontogenetic shape changes described in this study represent real observable differences in cranial anatomy, they exist within the context of taphonomic deformation, and should always be considered within this framework.

## 5. Conclusion

Using three-dimensional cranial landmarks from an ontogenetic series of *Diictodon*, we described the allometric changes in cranial shape. We showed that the effects of sexual dimorphism on the population are negligible and readily identifiable differences in allometric growth are possibly constrained only to the region of the caniniform process. Our results have revealed different allometric trajectories for each morphotype, and we discuss the potential bias introduced into the data when morphotypes cannot be accurately classified from gross morphology. These findings raise important questions about the timing and plasticity of tusk eruption and add to a growing body of evidence that beyond the caniniform, dimorphic traits are negligible. More specifically this study revealed that *Diictodon* crania undergo significant morphological changes through ontogeny, which are probably associated with the development of increased muscular support for the jaw to accommodate a dietary shift later in ontogeny that permitted the consumption of larger volumes of tougher plant material—a pattern which is reflected in extant reptiles. Our results indicate that *Diictodon*’s cranial allometry conforms to the widespread evolutionary growth pattern known as CREA, and suggests that there may be evidence for the emergence or convergence of CREA in Anomodontia. In our analysis, we demonstrated that analysis of similarly deformed specimens can reduce the influence of taphonomic deformation and aid in the detection of morphological signals. We postulate that the non-allometric component of variation in the *Diictodon* sample represents a combination of natural variation in cranial shape (perhaps plasticity) and artefacts of deformation that are not uniaxial. Future work would benefit from improved strategies to handle the asymmetric component of taphonomic deformation that could not be accounted for in this study, which could assist in delimiting the boundaries of population variation in cranial shape.

## Data Availability

Supplementary material is available at Figshare [93].
